# Body weight definitions for evaluating a urinary diagnosis of acute kidney injury in patients with sepsis

**DOI:** 10.1186/s12882-018-0895-4

**Published:** 2018-05-02

**Authors:** Shinshu Katayama, Kansuke Koyama, Yuya Goto, Toshitaka Koinuma, Ken Tonai, Jun Shima, Masahiko Wada, Shin Nunomiya

**Affiliations:** Division of Intensive Care, Department of Anesthesiology and Intensive Care Medicine, Jichi Medical University School of Medicine, 3311-1, Yakushiji, Shimotsuke, Tochigi, 329-0498 Japan

**Keywords:** Acute kidney injury, Body mass index, Cystatin C, Ideal body weight, Intensive care unit, Sepsis

## Abstract

**Background:**

We hypothesized that the use of actual body weight might lead to more frequent misdiagnosis of acute kidney injury (AKI) than when ideal body weight is used in underweight and/or obese patients. We examined which definition of body weight is most effective in establishing a urinary diagnosis of AKI in septic patients.

**Methods:**

Consecutive patients aged ≥ 20 years admitted to the intensive care unit of a university hospital between June 2011 and December 2016 were analyzed. Sepsis was defined in accordance with the Sepsis-3 criteria. AKI was defined as a urinary output of < 0.5 mL/kg/6h during intensive care unit stay. Patients were divided into one of four body mass index-based classes. The severity of illness and 90-day mortality were compared across the body mass index subgroups in patients diagnosed using the actual body weight or ideal body weight.

**Results:**

Of 5764 patients, 569 septic patients were analyzed. One hundred and fifty-three (26.9%) and 140 (24.6%) patients were diagnosed as having AKI using actual body weight and ideal body weight, respectively. There were no significant differences in the severity of illness among these groups. Also, 90-day mortality did not differ significantly among these groups. According to body mass index, 90-day mortality significantly differed in patients diagnosed using their actual body weights (underweight vs. normal vs. overweight vs. obese: 76.7% vs. 39.5% vs. 26.0% vs. 35.7%, *P* = 0.033).

**Conclusion:**

Generally, using actual body weight to calculate the weight-adjusted hourly urine output for diagnosing AKI increased the sensitivity compared to ideal body weight, irrespective of the severity of illness in septic patients. Delayed diagnosis, however, was more common among underweight patients in this situation, and clinicians should be cautious when diagnosing urinary AKI using actual body weight.

## Background

Acute kidney injury (AKI) is a major complication in sepsis, and is associated with a high mortality rate. Nearly half of the patients in the intensive care unit (ICU) develop AKI, and the mortality rate among these patients ranges from 30% to 50% [1, 2]. Early recognition of AKI is therefore important as it allows for early treatment initiation and better prognostication of the clinical course in patients with sepsis.

The definition of AKI is based on absolute or relative changes in serum creatinine levels and the weight-adjusted hourly urine output [3]. The weight-adjusted hourly urine output is closely associated with early onset of AKI and high mortality rates [4–6]. To date, however, the precise body weight definition that should be used for establishing a urinary diagnosis of AKI remains unclear. For example, in patients with obesity or weight loss, there is a substantial difference between the actual body weight (ABW) and ideal body weight (IBW), which can lead to under- or over-diagnosis of AKI.

Only one study [7] has evaluated the influence of body weight on the urinary diagnosis of AKI thus far; they reported that ABW was more sensitive but less specific than IBW. In contrast, in acute respiratory distress syndrome (ARDS) [8] and obese patients [9], IBW is recommended over ABW because the sizes of the vital organs are more closely related to IBW than to ABW. In this regard, we hypothesized that IBW is more useful in terms of evaluating renal function and establishing a urinary diagnosis of AKI than ABW in heterogeneous patient populations.

Therefore, in this study, we investigated the influence of body weight definitions on the urinary diagnosis of AKI in septic patients who were at risk of AKI. In addition, we evaluated the severity of illness and 90-day mortality for each body weight type determined according to each patients’ body mass index (BMI).

## Methods

### Study design and setting

This was a single-center, retrospective, observational study that was conducted in a 14-bed general ICU of a university hospital (Tochigi, Japan). Clinical decisions were made at the discretion of the attending ICU physicians. The Institutional Research Ethics Committee of Jichi Medical University Hospital approved this study and waived the need for informed consent because of the retrospective nature of the study (No. 16–116).

### Participants

Data for consecutive patients admitted to the ICU between June 2011 and December 2016 were screened. Patients were included in the study if they were ≥ 20 years of age, admitted to the ICU for a minimum of 6 continuous hours, underwent hourly urine output measurements, and were diagnosed with sepsis according to the Third International Consensus Definitions for Sepsis and Septic Shock (Sepsis-3) [10]. Patients with end-stage renal failure who were on chronic dialysis, those with inadequate hourly urine output measurements, and those with missing data for body weight and/or height were excluded.

### Definitions

The urinary diagnosis and staging of AKI were based on the Kidney Disease: Improving Global Outcomes (KDIGO) criteria [1]. A urinary diagnosis of AKI is established via 2 methods: the use of ABW or IBW for the calculation of weight-adjusted hourly urine output; in this study, these factors are indicated as AKI [ABW] and AKI [IBW], respectively. ABW was measured on or around the day of ICU admission. IBW was calculated using the Devine formula as follows [11]:

Males: IBW (kg) = 50 kg + 0.91 * (Height [cm] – 152.4).

Females: IBW (kg) = 45.5 kg + 0.91 * (Height [cm] – 152.4).

Patients were classified into one of four BMI classes: underweight (BMI < 18.5), normal (18.5 ≤ BMI < 25), overweight (25 ≤ BMI < 30), and obese (30 ≤ BMI). Chronic kidney disease (CKD) was defined as an estimated glomerular filtration rate (eGFR) of < 60 mL/min/1.73 m^2^ [12]. Overt disseminated intravascular coagulation was defined according to the criteria of the International Society on Thrombosis and Haemostasis [13]. The baseline serum creatinine value was defined as the stable value observed over the year prior to hospital admission. For patients whose baseline serum creatinine values were unknown, we used the Modification of Diet in Renal Disease (MDRD) equation, and assumed a baseline eGFR of 75 mL/min/1.73 m^2^ [14].

### Data collection

In this study, we collected the urine output data during the ICU stay (maximum of 7 days). The following information was collected for all patients: age; sex; body weight; body height; premorbid creatinine level; infection site (intra-cranial, head and neck, thoracic, abdominal, urinary tract, skin and soft tissue, catheter related blood stream infection, or others); ischemic heart disease, chronic heart failure, hypertension, arrhythmia, chronic obstructive pulmonary disease, cerebrovascular accident, diabetes mellitus, hepatic disease, or chronic kidney disease; an immunocompromised state; use of aminoglycoside or vancomycin; the Simplified Acute Physiology Score II (SAPS-II) [15]; and the Sequential Organ Failure Assessment (SOFA) score [16]. We also recorded the presence of septic shock, overt disseminated intravascular coagulation, the requirement of mechanical ventilation, duration of mechanical ventilation and ICU stay, and mortality rate at 90 days. In our ICU, hourly urine output measurements are made for almost all patients, except for patients undergoing renal replacement therapy (RRT) whose urine outputs are measured every 3 h. Therefore, in the case of RRT patients, we calculated the mean hourly urine output. The highest values of serum creatinine and cystatin C levels were mostly realized in the first 48 h after admission to the ICU.

### Statistical analyses

Variables were compared between the two groups using Fisher’s exact test, Pearson’s chi-squared test, and the Mann–Whitney *U* test as appropriate. The Wilcoxon/Kruskal-Wallis test was used to compare variables among BMI classes. The Kaplan-Meier method and log-rank test were used to calculate the cumulative mortality rate. The risk ratio of mortality was calculated using a proportional hazard model. Multiple regression lines were analyzed to evaluate the relationships between cystatin C and creatinine values across BMI classes. All analyses were performed using the JMP 13 software program (SAS Institute Inc., Cary, NC, USA). The data are presented as medians and interquartile ranges (25th–75th percentiles) or percentages. *P*-values less than 0.05 were considered statistically significant.

## Results

### Enrollment and baseline characteristics

**Fig. 1:**
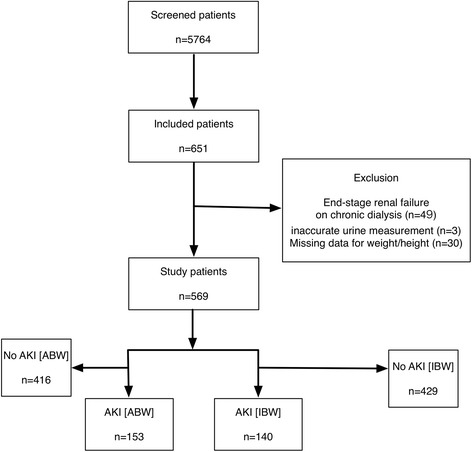
Flow chart of this study. ABW, actual body weight; AKI, acute kidney injury; BW, body weight; CKD, chronic kidney disease; IBW, ideal body weight

During the study period, 5764 patients were admitted to the ICU. Among these, 651 were diagnosed with sepsis; furthermore, 49 patients who were receiving chronic dialysis owing to end-stage renal failure, 3 with inaccurate urine measurements (i.e., bladder irrigation), and 30 with missing body weight and height measurements were excluded. Thus, data for a total of 569 patients were analyzed in this study (Fig. 1).

The characteristics of the analyzed patients are summarized in Table 1. Herein, 153 (26.9%) and 140 (24.6%) patients were diagnosed with AKI [ABW] and AKI [IBW], respectively. The mean (range) BMI of the AKI [ABW] and AKI [IBW] groups were 23.6 (21.1–26.6) and 23.0 (20.1–25.5), respectively. Baseline characteristics were comparable between the groups. Besides, the severity of illness between groups did not differ significantly, and 90-day mortality was 38.4% in the AKI [ABW] group and 42.3% in the AKI [IBW] group (Table 1).

**Table 1:** Patient characteristics

Characteristics	Total	AKI [ABW]	AKI [IBW]	*P*-value
	*n* = 569	*n* = 153	*n* = 140	
Age, years	69 (59–78)	69 (59–78)	69 (59–78)	0.998
Male sex, No.	305 (53.6%)	89 (58.2%)	89 (63.6%)	0.344
Body weight, kg	57 (48–66)	59 (52–70)	58 (50–68)	0.293
Height, cm	158 (150–166)	160 (152–167)	160 (154–167)	0.452
BMI, kg/m^2^	22.5 (19.7–25.3)	23.6 (21.1–26.6)	23.0 (20.1–25.5)	0.082
Infection site, No.				0.999
CNS	6 (1.0%)	0 (0%)	0 (0%)	
Thorax	128 (22.5%)	37 (24.2%)	33 (23.6%)	
Abdomen	301 (52.9%)	72 (47.1%)	64 (45.7%)	
Head and neck	25 (4.4%)	1 (0.7%)	1 (0.7%)	
Soft tissue	32 (5.6%)	14 (9.2%)	13 (9.3%)	
UTI	25 (4.4%)	2 (1.3%)	2 (1.4%)	
CR-BSI	5 (0.9%)	4 (2.6%)	3 (2.1%)	
Other	49 (8.6%)	23 (15.0%)	24 (17.1%)	
Unknown premorbid creatinine, No.	254 (44.6%)	58 (37.9%)	54 (38.6%)	
Premorbid creatinine, mg/dL	0.75 (0.60–0.96)	0.82 (0.63–1.09)	0.83 (0.69–1.18)	0.756
Comorbidities, No.				
CKD	158 (27.8%)	58 (61.1%)	54 (61.4%)	0.810
IHD	53 (9.3%)	18 (11.8%)	14 (10.0%)	0.629
CHF	50 (8.8%)	21 (13.7%)	16 (11.4%)	0.554
Hypertension	273 (48.0%)	84 (54.9%)	74 (52.9%)	0.726
Arrhythmia	57 (10.0%)	23 (15.0%)	20 (14.3%)	0.857
COPD	32 (5.6%)	6 (3.9%)	7 (5.0%)	0.654
CVA	62 (10.9%)	21 (13.7%)	16 (11.4%)	0.554
DM	145 (25.5%)	47 (30.7%)	43 (30.7%)	0.999
Immune dysfunction	171 (30.0%)	54 (35.3%)	51 (36.4%)	0.840
Hepatic disease	53 (9.3%)	18 (11.8%)	18 (12.9%)	0.776
Aminoglycoside use	8 (1.4%)	3 (2.0%)	3 (2.1%)	0.913
Vancomycin use	139 (24.4%)	55 (36.0%)	49 (35.0%)	0.866
Septic shock	247 (43.4%)	87 (56.9%)	81 (57.9%)	0.864
ISTH DIC	125 (22.5%)	55 (36.7%)	54 (39.4%)	0.632
SOFA score	7 (4–9)	9 (7–11)	10 (7–12)	0.617
SAPS II	50 (39–62)	63 (52–77)	64 (51–77)	0.760
Urinary diagnosis of AKI, No.				0.852
Stage 1		51 (9.0%)	45 (7.9%)	
Stage 2		38 (6.7%)	32 (5.6%)	
Stage 3		64 (11.2%)	63 (11.1%)	
Mechanical ventilation, No.	462 (81.2%)	139 (90.9%)	124 (88.6%)	0.521
Days of mechanical ventilation	7 (5–12)	8 (6–15)	9 (6–16)	0.957
Days of ICU stay	8 (5–12)	10 (7–16)	10 (6–16)	0.862
90-day mortality	19.0%	38.4%	42.3%	0.624

*ABW* actual body weight, *AKI* acute kidney injury, *BMI* body mass index, *BW* body weight, *CHD* chronic heart disease, *CKD* chronic kidney disease, *CNS* central nervous system, *COPD* chronic obstructive pulmonary disease, CR-BSI catheter-related blood stream infection, *CVA* cerebrovascular accident, *DIC* disseminated intravascular coagulation, *DM* diabetes mellitus, *IBW* ideal body weight, *ICU* intensive-care unit, *ISTH* International Society on Thrombosis and Hemostasis, *SAPS II* sequence assessment of physiological score II, *SOFA* Sequential Organ Failure Assessment, *UTI* urinary tract infection

In the AKI [ABW] group, 129 (84.3%) patients were diagnosed with AKI based on the level of serum creatinine, and 24 (15.7%) without AKI. On the other hand, in the AKI [IBW] group, 122 (87.1%) patients were diagnosed with AKI and 18 (12.9%) without AKI based on the level of serum creatinine.

### Urinary diagnosis of AKI and staging with ABW and IBW

In the AKI [ABW] group, 51 (9.0%) patients had stage 1 disease, 38 (6.7%) stage 2, and 64 (11.3%) stage 3. In the AKI [IBW] group, 45 (7.9%) patients had stage 1 disease, 32 (5.6%) stage 2, and 63 (11.1%) stage 3. The concordance rate between the groups was 92.4% (*n* = 526). When using ABW to diagnose AKI, 31 patients (5.4%) were classified into higher AKI stages, while 12 patients (2.2%) were classified into lower AKI stages than those diagnosed using IBW (Table 2).

**Table 2:** Acute kidney injury diagnoses and staging

		AKI [ABW]				
		Stage 0	Stage 1	Stage 2	Stage 3	Total
AKI [IBW]	Stage 0	406	20	2	1	429
		71.4%	3.5%	0.4%	0.2%	75.5%
	Stage 1	8	29	8	0	45
		1.4%	5.1%	1.4%	0%	7.9%
	Stage 2	2	2	28	0	32
		0.4%	0.4%	4.9%	0%	5.7%
	Stage 3	0	0	0	63	63
		0%	0%	0%	11.1%	11.1%
	Total	416	51	38	64	
		73.1%	9.0%	6.7%	11.3%	

Upper: Number of patients

Lower: Percentage

*ABW* actual body weight, *AKI* acute kidney injury, *IBW* ideal body weight

### Relationship between urinary diagnosis of AKI and BMI

Table 3 shows the relationship between urinary diagnosis of AKI and BMI. The AKI [ABW] group included 12 underweight patients (7.8%), 83 patients (54.2%) with normal weight, 40 overweight patients (26.1%), and 18 obese patients (11.8%). The AKI [IBW] group comprised 19 underweight patients (13.6%), 80 patients (57.1%) with normal weight, 28 overweight patients (20.0%), and 13 obese patients (9.3%).

**Table 3:** Characteristics of body mass index subgroups in the acute kidney injury [actual body weight] and [ideal body weight] groups

Group	AKI [ABW]					AKI [IBW]				
Subgroup	Under weight	Normal	Over weight	Obese	*P*-value	Under weight	Normal	Over weight	Obese	*P*-value
Patients (n)	12	83	40	18		19	80	28	13	
Age, years	60 (53–69)	69 (62–78)	73 (64–79)	60 (44–72)	0.006	66 (53–77)	69 (61–78)	75 (66–80)	59 (46–70)	0.015
Male sex, No.	7 (58.4%)	51 (61.5%)	20 (50.0%)	11 (61.1%)	0.676	12 (63.1%)	52 (65.0%)	16 (57.1%)	9 (69.2%)	0.861
Body weight, kg	42 (40–48)	55 (50–60)	67 (58–73)	90 (76–95)	< 0.0001	43 (40–49)	58 (51–61)	67 (58–73)	92 (80–97)	< 0.0001
Height, cm	159 (151–175)	160 (153–166)	156 (147–166)	164 (154–170)	0.283	160 (150–174)	160 (155–167)	156 (147–166)	165 (160–171)	0.103
BMI, kg/m^2^	16.9 (16.0–17.8)	22.1 (20.3–23.6)	26.6 (25.8–27.9)	32.4 (31.3–38.2)	< 0.0001	17.1 (16.0–17.8)	22.1 (20.8–23.6)	26.5 (25.5–27.8)	32.1 (30.8–34.2)	< 0.0001
Premorbid creatinine, mg/dL	0.74 (0.56–0.86)	0.84 (0.70–1.25)	0.77 (0.61–1.06)	0.89 (0.63–1.23)	0.332	0.75 (0.56–0.91)	0.85 (0.70–1.29)	0.78 (0.67–1.06)	0.96 (0.60–1.31)	0.433
Highest creatinine level within 48 h, mg/dL	1.83 (1.08–2.61)	2.27 (1.16–4.18)	2.13 (1.11–3.07)	2.53 (1.22–4.09)	0.366	1.51 (0.84–2.59)	2.31 (1.36–4.20)	2.56 (1.53–3.60)	2.92 (2.10–5.50)	0.018
Highest cystatin C level within 48 h, ng/dL	2.78 (2.32–3.64)	2.31 (1.49–3.36)	1.74 (1.01–2.58)	1.97 (1.29–4.78)	0.036	2.46 (1.00–3.08)	2.27 (1.46–3.36)	1.92 (1.58–3.05)	2.85 (1.97–4.40)	0.596
SAPS II	65 (46–83)	63 (52–75)	64 (55–76)	58 (45–78)	0.886	55 (44–77)	64 (51–75)	67 (60–81)	62 (49–81)	0.105
SOFA score	10 (7–14)	9 (6–11)	8 (6–11)	11 (8–13)	0.406	7 (4–12)	9 (7–11)	11 (7–13)	12 (9–13)	0.082
ISTH DIC, No.	45.5%	41.5%	28.2%	27.8%	0.393	33.3%	45.6%	29.6%	30.8%	0.388
Septic shock, No.	66.7%	56.6%	50.0%	66.7%	0.584	42.1%	58.8%	60.7%	69.2%	0.433
Urinary diagnosis of AKI, No.					0.446					0.439
Stage 1	3 (25.0%)	26 (31.3%)	17 (42.5%)	5 (27.8%)		8 (42.1%)	24 (30.0%)	9 (32.1%)	4 (30.8%)	
Stage 2	4 (33.3%)	17 (20.5%)	12 (30.0%)	5 (27.8%)		6 (31.6%)	17 (21.3%)	8 (28.6%)	1 (7.7%)	
Stage 3	5 (41.7%)	40 (48.2%)	11 (27.5%)	8 (44.4%)		5 (26.3%)	39 (48.8%)	11 (39.3%)	8 (61.5%)	
MV, No.	83.3%	86.8%	100.0%	94.4%	0.078	79.0%	86.3%	100.0%	92.3%	0.114
Duration of MV, day	8 (6–13)	8 (6–16)	11 (6–14)	8 (5–16)	0.803	10 (7–12)	9 (6–16)	11 (6–20)	7 (4–15)	0.535
Days of ICU stay	11 (6–12)	9 (7–16)	13 (8–17)	10 (5–18)	0.429	11 (5–13)	9 (6–16)	13 (7–22)	8 (4–16)	0.272
90-day mortality	76.7%	39.5%	26.0%	35.7%	0.033	49.2%	40.8%	37.8%	48.7%	0.736

*ABW* actual body weight, *AKI* acute kidney injury, *BMI* body mass index, *DIC* disseminated intravascular coagulation, *IBW* ideal body weight, *ICU* intensive care unit, *ISTH* International Society on Thrombosis and Hemostasis, *MV* mechanical ventilation, *SAPS II* sequence assessment of physiological score II, *SOFA* Sequential Organ Failure Assessment, *UTI* urinary tract infection

In the AKI [ABW] group, the underweight BMI subgroup demonstrated significantly higher mortality rates than the other BMI subgroups (*P* = 0.033), although the severity of illness (i.e., SAPS II and SOFA score) was not significantly different. However, in the AKI [IBW] group, the mortality rate was not significantly different among BMI subgroups (*P* = 0.736; Table 3). The risk ratio of mortality in the underweight subgroup of the AKI [ABW] group was 2.62 (95% confidence interval [CI] 1.17–5.32, *P* = 0.021) when compared with the normal subgroup, 4.04 (95% CI 1.62–9.79, *P* = 0.0035) when compared with the overweight subgroup, and 1.98 (95% CI 0.77–5.10, *P* = 0.152) when compared with the obese subgroup (Table 4).

**Table 4:**
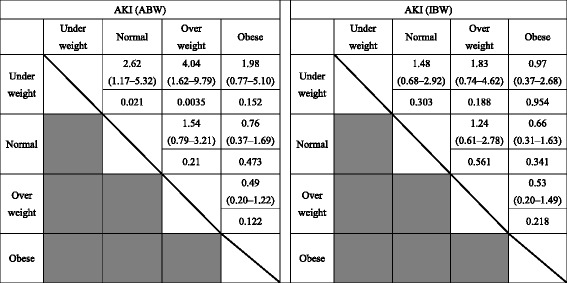
Risk ratio for cumulative mortality according to BMI subgroups

Upper: Risk ratio (95% confidence interval)

Lower: *P*-value

*ABW* actual body weight; *AKI* acute kidney injury; *BMI* body mass index; *IBW* ideal body weight

### Relationship between cystatin C and creatinine among the BMI subgroups

Table 3 shows serum creatinine levels according to the BMI classification in the AKI [ABW] and AKI [IBW] groups. In the AKI [IBW] group, the highest serum creatinine levels significantly increased in line with the BMI (underweight, 1.51 [0.84–2.59]; normal, 2.31 [1.36–4.20]; overweight, 2.56 [1.53–3.60]; and obese, 2.92 mg/dL [2.10–5.50], *P* = 0.018). However, in the AKI [ABW] group, creatinine levels did not significantly differ across BMI subgroups (*P* = 0.366).

On the other hand, in the AKI [ABW] group, serum cystatin C levels differed significantly across the BMI subgroups (*P* = 0.036). In particular, the underweight and normal subgroups showed significantly higher cystatin C levels than the overweight group (2.78 [2.32–3.64] vs. 1.74 [1.01–2.58] ng/mL, *P* = 0.023; 2.31 [1.49–3.36] vs. 1.74 [1.01–2.58] ng/mL, *P* = 0.015, respectively). However, in the AKI [IBW] group, serum cystatin C levels did not differ significantly among the BMI subgroups, and they also showed a homogeneous distribution (*P* = 0.596).

## Discussion

Body weight must be taken into consideration in various clinical situations, such as in the management of ARDS and administration of drugs. For example, the low tidal ventilation strategy as a management approach to ARDS is selected based on the IBW, because the lung volume is more closely related to the IBW than the ABW [17, 18]. In contrast, when calculating the dosage of medications for morbidly obese patients, there is no marked difference in the utility between ABW and IBW [9]. Furthermore, fluid resuscitation is crucial in the management of patients with sepsis, and can increase the body weight by up to 10% after initial resuscitation [19]. In such cases, it is unclear whether we should determine the baseline body weight at ICU admission or after the patient’s condition has stabilized a few hours later. In contrast, IBW can be estimated based on the sex and height, which are not affected by fluid balance. Furthermore, renal function is not affected by changes in temporary variables such as daily body weight. As such, the use of IBW seems logically more suitable for evaluating renal function and establishing a urinary diagnosis of AKI than ABW.

In this retrospective study, we investigated the most appropriate body weight definition required to accurately make a urinary diagnosis of AKI. We found a discrepancy rate of 7.6% in terms of the urinary diagnosis of AKI between AKI [ABW] and AKI [IBW]. The number cases of AKI [ABW] was higher than AKI [IBW], which meant a higher sensitivity for detecting AKI. In the AKI [ABW] group, the mortality rate was significantly different among the BMI subgroups, while in the AKI [IBW] group, there were no significant differences in the mortality rates among the BMI subgroups. However, there was no difference in the severity of illness among patients diagnosed with AKI based on ABW or IBW. In this regard, the type of body weight might not have clinical significance in terms of the early recognition of urinary AKI, although AKI [ABW] presented higher sensitivity than AKI [IBW].

To date, there is no consensus about what type of body weight should be used to calculate the weight-adjusted hourly urine output for the urinary diagnosis of AKI according to the KDIGO criteria. Only one study has examined this issue [7]; that study found that ABW was more sensitive but less specific than IBW in terms of diagnosing and staging AKI using the urine output criterion. However, the population in that study had a higher mean BMI than our studied population (28.0 vs. 22.5), and only 7.8% of patients had a BMI ≤ 20. Therefore, especially for underweight patients, the utility of ABW to diagnose AKI with regards to its superior sensitivity when compared to IBW remains unclear; our findings suggest that AKI [ABW] might yield more heterogeneity in terms of mortality rate prediction and timing of recognizing AKI than AKI [IBW]. In this study, however, there were no significant differences between the two groups in severity of illness. Previous studies suggested that overweight and obese patients presented lower mortality than underweight patients irrespective of the severity of illness [20, 21]. From this perspective, increased mortality in underweight patients with AKI [ABW] seems more of an “obesity paradox” rather than an underdiagnosis. In this regard, the types of body weight used for urinary AKI might have no clinical significance.

For the early recognition of AKI, 6-h urine output of < 0.5 mL/kg/h and elevated serum creatinine level (≥ 0.3 mg/dL) have been used [3]. Moreover, AKI could be more sensitively detected using both the urine output and creatinine criteria, than using the creatinine criteria alone [4–6, 22]. However, creatinine production is highly heterogeneous across individuals, differs according to muscle mass, physical activity, and dietary meat consumption, and tends to overestimate the prevalence of AKI in obese patients and underestimate the prevalence in underweight patients. In addition, the urinary diagnosis of AKI depends on the type of body weight used. In this context, the present AKI definition might not be ideal for patients with abnormal body weight such as morbidly obese patients. However, serum creatinine levels would easily be elevated in this population because of their high muscle mass, and this phenomenon could help to make an early diagnosis of AKI. Our study supported this speculation: serum creatinine levels increased in line with the BMI. Creatinine shows small variations in underweight patients because of their lower muscle mass than in obese patients, and this might lead to the under-recognition of AKI despite using two different definitions of AKI, creatinine, and urine output criteria.

Measurement of urine output is not only more sensitive and quicker than measuring serum creatinine once a day in detecting AKI, but it is also important to realize that patients only fulfilling AKI definitions based on urine output criteria differ from patients fulfilling AKI definitions based on serum creatinine with or without urine output measurements. In this study, 15.7% of AKI [ABW] patients and 12.9% of AKI [IBW] patients were not diagnosed with AKI based on the serum creatinine definition. In this regard, serum creatinine and urine output for diagnosing AKI do not always act complementarily, but may show independent pathophysiologies. Further studies are needed to evaluate this concern in these populations.

To evaluate renal function, serum creatinine was commonly used. However, its value was highly affected by body types. Recently, cystatin C has alternatively been recommended for the diagnosis of AKI instead of creatinine [23]. Cystatin C is a serum protein filtered freely at the glomerulus as is creatinine, but unlike creatinine, it is produced by all nucleated cells and its level is not determined by muscle mass; therefore, its generation appears to be more uniform across populations [24]. Generally, cystatin C is not recommended to evaluate renal function, and KDIGO guidelines still use serum creatinine. On the other hand, there are several studies in which cystatin C appears to be a better biomarker in the prediction of AKI, especially in the early phase [25–28]. The concentration of cystatin C peaks earlier than serum creatinine in patients with AKI, and may enable the earlier detection of kidney dysfunction than creatinine [29]. In this study, in line with previous studies, we used cystatin C to evaluate renal function along with serum creatinine in more detail. As a result, both underweight and overweight patients in the AKI [ABW] group showed highly heterogeneous serum cystatin C levels. In this regard, we should recognize that the use of ABW to establish a urinary diagnosis of AKI would result in delayed recognition of AKI in the underweight group, compared to the findings obtained using IBW, irrespective of their severity of illness. To confirm this finding, further studies are needed to evaluate the relationship between BMI and cystatin C concentration in septic AKI patients.

### Limitations

Several limitations associated with the present study should be mentioned. First, this was a single-center, retrospective study. The sample size was relatively small, particularly when divided into BMI subgroups. Further studies are needed to confirm our findings, especially for the underweight patients. Second, we did not evaluate fluid balance before admission to the ICU; therefore, the baseline body weight might differ from that in the pre-morbid state. In addition, the timing of body weight measurement was not always just after ICU admission. However, we tried to select the most reliable body weight value after ICU admission in an effort to minimize any errors. Third, we did not measure other AKI biomarkers, such as urine neutrophil gelatinase-associated lipocalin (NGAL), to evaluate the accuracy of urinary AKI. Finally, whether or not the patients’ underweight statuses were due to malignant disease was unclear. The presence of such disease, for example, cachexia, might be associated with the relatively higher mortality rate observed in this subgroup than that in the other BMI subgroups.

Despite these limitations, our study has several strengths. All of our patients were evaluated using the Sepsis-3 criteria as being at risk for AKI. In addition, to the best of our knowledge, our study is the first to focus on various body weight types in order to evaluate the homogeneity of the timing of establishing a urinary diagnosis of AKI.

## Conclusion

Using ABW to calculate the weight-adjusted hourly urine output for diagnosing AKI generally increased the sensitivity compared with IBW irrespective of the severity of disease among both groups. This result could be expected due to the increasing number of overweight patients who earlier meet the definition of urinary AKI. Delayed diagnosis, however, was more common among underweight patients in this situation, and clinicians should be cautious when diagnosing urinary AKI using actual body weight.

## Abbreviations

ABW: Actual body weight
AKI: Acute kidney injury
ARDS: Acute respiratory distress syndrome
BMI: Body mass index
eGFR: Estimated glomerular filtration rate
IBW: Ideal body weight
ICU: Intensive care unit
KDIGO: The Kidney Disease Improving Global Outcomes
SAPS-II: Simplified Acute Physiology Score II
SOFA: Sequential Organ Failure Assessment
